# Effects of Choline on Meat Quality and Intramuscular Fat in Intrauterine Growth Retardation Pigs

**DOI:** 10.1371/journal.pone.0129109

**Published:** 2015-06-05

**Authors:** Bo Li, Wei Li, Hussain Ahmad, Lili Zhang, Chao Wang, Tian Wang

**Affiliations:** College of Animal Science and Technology, Nanjing Agricultural University, Nanjing, Jiangsu, China; Wageningen UR Livestock Research, NETHERLANDS

## Abstract

The aim of this study was to investigate the effects of choline supplementation on intramuscular fat (IMF) and lipid oxidation in IUGR pigs. Twelve normal body weight (NBW) and twelve intrauterine growth retardation (IUGR) newborn piglets were collected and distributed into 4 treatments (Normal: N, Normal+Choline: N+C, IUGR: I, and IUGR+Choline: I+C) with 6 piglets in each treatment. At 23 d of age, NBW and IUGR pigs were fed basal or choline supplemented diets. The results showed that the IUGR pigs had significantly lower (*P*<0.05) BW as compared with the NBW pigs at 23 d, 73 d, and 120 d of age, however, there was a slight decreased (*P*>0.05) in BW of IUGR pigs than the NBW pigs at 200 d. Compared with the NBW pigs, pH of meat *longissimus dorsi* muscle was significantly lower (*P*<0.05), and the meat color was improved in IUGR pigs. The malondialdehyde (MDA) levels were significantly decreased (*P*<0.05), while triglyceride (TG) and IMF contents were significantly higher (*P*<0.05) in the IUGR pigs than the NBW pigs. IUGR up-regulated the *m*RNA gene expression of fatty acid synthetase (FAS) and acetyl-CoA carboxylase (ACC). Dietary choline significantly increased (*P*<0.05) the BW at 120d of age, however, significantly decreased (*P*<0.05) the TG and IMF contents in both IUGR and NBW pigs. FAS and sterol regulatory element-binding proteins 1 (SREBP1) *m*RNA gene expressions were increased (*P*<0.05) while the muscle-carnitine palmityl transferase (M-CPT) and peroxisome proliferators-activated receptorγ (PPARγ) *m*RNA (*P*<0.05) gene expressions were decreased in the muscles of the IUGR pigs by choline supplementation. Furthermore, choline supplementation significantly increased (*P*<0.05) the MDA content as well as the O_2_•¯ scavenging activity in meat of IUGR pigs. The results suggested that IUGR pigs showed a permanent stunting effect on the growth performance, increased fat deposition and oxidative stress in muscles. However, dietary supplementation of choline improved the fat deposition via enhancing the lipogenesis and reducing the lipolysis.

## Introduction

Developmental plasticity defined the phenomenon by which one genotype can lead to a range of different physiological or morphological states in response to different environmental conditions during development. This concept was introduced following epidemiological long-term studies in humans fed different diets in early life [1], especially in infants suffering from intra-uterine growth retardation [2]. IUGR defined as impaired growth and development of the mammalian embryo or its organs during pregnancy, which is a major concern in domestic animal production, it can easily cause low birth weight, damage gastrointestinal function and reduce neonatal survival [3]. Epidemiological studies show that the IUGR adults are more prone to suffer from obesity and other metabolic syndromes. Nowadays, pigs have been used as the model animal in physiological, nutritional and metabolic studies as compared with human. Pigs, sharing many physiological similarities with humans, offers several breeding and handling advantages, making it an optimal species for preclinical experimentation [4,5], such as, the effects of surgical techniques in the gastrointestinal tract were studied in a pig model and could explain the difference in recovery in patients [6] and the three-week-old piglet as a model animal for studying protein digestion in human infants [7]. Thus, pigs provide crucial prerequisites and ample opportunity for the development of safe preclinical protocols in biomedical studies and on the consequences of nutritional programming. Previous studies suggested that the IUGR may affect skeletal muscle metabolism leading to more fat deposition [8], as fat accumulation correlated with systemic oxidative stress [9,10] and it may have a long-term impairment negatively affecting on meat quality [11]. Puglianiello [12] also found that the IUGR could increase the gene expressions related to lipolysis, resulted in the suffering from oxidative stress in the IUGR pigs, therefore, it is essential to suppress obesity in meat production in the IUGR pigs.

Choline, a water-soluble vitamin, has been shown to have a positive effect on animal production [13,14], which plays a vital role in neurotransmission, membrane integrity and methylation pathways [15,16]. Previous studies shown that choline deficiency can cause pathological changes in many organs, such as the necrosis and cirrhosis of the liver, the risk of cancer, cortical necrosis and renal failure in kidney as well as memory degradation [17–19]. However, a little information is available on the intramuscular fat metabolism in IUGR pig models. Some data have suggested that choline attenuates oxidative stress in patients with asthma [17]. A previous study reports that the dietary choline could induce the oxidation [20,21] of lipid component in the membranes of muscle cells and also affected the intracellular lipid metabolism [22]. Therefore, this underlines the importance of choline-rich foods for proper muscle function. Our objectives were to investigate the role of dietary choline on growth performance, meat quality, antioxidant capacity of meat and intramuscular fat metabolism in IUGR pigs, which may make a sense or be a reference in humans especially in IUGR fetuses in humans.

## Materials and Methods

### Animal care

The experiments approved and conducted under the supervision of the Animal Care and Use Committee, Nanjing Agricultural University, Nanjing, People's Republic of China, which has adopted the Animal Care and Use Guidelines governing all animals used in experimental procedures.

### Experimental design

Twelve normal body weight (NBW) and twelve IUGR of Landrace×Large White piglets were collected from 12 litters according to Xu [23] who defined IUGR piglets body weight is less than two standard deviations of the average weight of piglets (birth weight range: NBW, 1.5 ± 0.2Kg: IUGR, 0.75 ± 0.2Kg). Each IUGR piglet had the sibling of NBW piglets. All piglets were freely breast fed by sows at 23d of age. Then twelve NBW and twelve IUGR piglets were randomly divided into following four groups and each group had six piglets. NBW piglets fed with basal diet (Group N), NBW piglets with choline supplementation (Group N+C), IUGR piglets fed with basal diet (Group I), and IUGR piglets with choline supplementation (Group I+C). After the period of weaning, all pigs were allowed water *ad libitum* while feed were fed three times per day at 06.30, 11.00, and 18.00 hours. Basal diets are shown in **Table 1**, which contained normal levels of choline (NRC, 1998). Choline chloride (50% choline chloride; Shandong Enbei Group Ltd., P.R.China) was used as a dietary supplementation alternative to the same amount of corn; its choline content was two times higher than the basal diets (**Table 2**).

**Table 1:** Composition of basal diet, as-fed basis.

Item	Growth stage			
	23–35 d	38–73 d	74–120 d	121–200 d
Ingredient,%				
Corn	64	65	60	68
Soybean meal	26	25	26	24
Fish meal	4	2	0	0
Wheat bran	2	4	4	4
Wheat grain	0	0	16	0
L-Lys·HCl	0.25	0.15	0.15	0.15
CaHPO4	0.5	0.5	0.55	0.5
Limestone	0.8	0.9	0.85	0.9
Salt	0.35	0.35	0.35	0.35
Premix ^*^	2.1	2.1	2.1	2.1
Calculated composition				
ME,kcal/kg	3700	2970	2996	2985
CP,%	21.7	18.7	15.3	17.3
Lys,%	1.68	1.07	0.76	0.95
Ca,%	1.06	0.61	0.52	0.53
TP,%	0.84	0.60	0.52	0.53

*ME* metabolic energy; *CP* crude protein; *TP* total phosphorus

*Premix per kilogram of diet: 8,800 IU vitamin A; 1,600 IU vitamin D; 40 mg vitamin E; 0.4 mg vitamin B_1_; 3.6 mg vitamin B_2_; 1 mg vitamin B_6_; 16 mg niacin,; 11 mg pantothenic acid; 180 mg choline chloride; 96 mg Cu as CuSO_4_; 88 mg Fe as FeSO_4_; 76 mg Zn as ZnSO_4_; 24 mg Mn as MnSO_4_; 0.4 mg Co as CoCl_2_; and 0.4 mg I as CaIO_3_.

**Table 2:** Choline addition and levels in diet.

Item,g/kg diet	23–37 d		38–73 d		74–120 d		120-200d	
	B	C	B	C	B	C	B	C
50% Choline chloride addition	0	3.60	0	3.23	0	3.12	0	3.03
Calculated level								
Choline addition	0	1.34	0	1.20	0	1.16	0	1.13
Total choline	1.34	2.68	1.20	2.40	1.16	2.32	1.13	2.26
Analyzed level								
Choline	1.59	2.98	1.33	2.77	1.40	2.71	1.40	2.75
Choline chloride	0.08	1.73	0.24	1.56	0.25	1.50	0.25	1.45

B = basal diet; C = diet with choline

The total choline content was calculated according to Feed Composition and Nutritive Values in the database website of China Feed (22nd rev) [24].

### Sample collection

Body weight was weighed at 23, 73, 120 and 200 d of age. At the age of 200 d, four pigs were randomly selected from each group and were slaughtered via electrical stunning followed by exsanguinations. Left *longissimus dorsi* (*LD*) meat muscles samples (5 g) were rapidly collected and washed with normal saline, and stored at -20°C for biochemical analysis, while, another piece of 2 g of *LD* meat muscle samples were immediately placed in liquid nitrogen stored at -80°C until further analysis.

### Determination of meat quality in longissimus dorsi muscle

Meat quality of *LD* meat muscle samples was evaluated by measuring drip loss (%), pH value and meat color. For drip loss, meat samples were weighed and suspended at 4°C, then re-weighed at 24 h and 48 h. pH values were measured at 1 h and 24 h by HI 9025 pH meter (Hanna instruments, Italy). Meat color was determined using a chromameter (Konica Minolta Sensing, Inc., Japan), and the results were described as L*, a* and b* (where L* measures relative lightness, a* relative redness, and b* relative yellowness). The average of the three measurements was used to calculate the hue angle (*H*; which represents the relative position of color between redness and yellowness; H* = 180/π arctan(b*/a*) and Chroma (*C*; the color intensity; C* = (*a* ^2^+ *b* ^2^)^1 /2^).

### Determination of superoxide, DPPH, and ABTS•+ radical scavenging activities and MDA levels in longissimus dorsi muscle

Superoxide radical (O_2_^•-^) scavenging activity and MDA levels were measured using the kits (Nanjing Jiancheng Bioengineering Institute, P.R. China); the spectrophotometric analysis of 2,2'-Azinobis-(3-ethylbenzthiazoline-6-sulphonate) radical cation (ABTS^•+^) scavenging activity was determined according to the method of Re [25]. 1,1-Diphenyl-2-picrylhydrazyl radical 2,2-Diphenyl-1-(2,4,6-trinitrophenyl)hydrazyl (DPPH) radical scavenging activity was measured according to the method of Cheng [26].

### Determination of TG and IMF content in longissimus dorsi muscle

The triglyceride (TG) content in muscles was measured using kits (Nanjing Jiancheng Bioengineering Institute, China) which was based on the method of GPO-PAP (Glycerol phosphate oxidase-Peroxidase and Anti Peroxidase). Intramuscular fat of samples was assessed by Soxhlet extraction.

### Determination of related mRNA gene expression of fat metabolism in longissimus dorsi muscle

Intramuscular fat related genes, including fatty acid synthetase (FAS), acetyl-CoA carboxylase (ACC), muscle-carnitine palmityl transferase (M-CPT), peroxisome proliferators-activated receptorγ (PPARγ) and sterol regulatory element-binding proteins (SREBP1) *m*RNA gene expressions were measured by real-time PCR (Applied Biosystems, USA). Primers (**Table 3**) for the assayed genes and the reference gene were designed using Primer Express 5.0 (Applied Biosystems).

**Table 3:** Primer sequences used in the RT-PCR.

Genes	Accession no.	Primers	Sequences (5`→3`)	bp
β-actin	XM003357928	Forward	GCGGGACATCAAGGAGAAG	216
		Reverse	GTTGAAGGTGGTCTCGTGG	
FAS	NM001099930	Forward	GCCTAACTCCTCGCTGCAAT	196
		Reverse	TCCTTGGAACCGTCTGTGTTC	
ACC	NM001114269	Forward	ATGTTTCGGCAGTCCCTGAT	133
		Reverse	GTGGACCAGCTGACCTTGA	
SREBP1	NM_214157	Forward	ACAGCCAGATGAAGCCAGAG	113
		Reverse	CAAGGGGTTGCAGGAGAGAC	
PPARγ	NM_214379	Forward	CCATTCCCGAGAGCTGATCC	93
		Reverse	GGACACAGGCTCCACTTTGA	
M-CPT1	NM001007191	Forward	AGCCAGATTGCCCAATTCCA	123
		Reverse	CGCGATCATGTAGGAGACCC	

All these primer sequences were designed based on the accession numbers described above.

FAS fatty acid synthetase, M-CPT1 muscle-carnitine palmityl transferase 1, ACC acetyl-CoA carboxylase, PPARγ Peroxisome proliferators-activated receptor γ, SREBP1, sterol regulatory element-binding proteins.

Total RNA of muscle tissues was extracted by using TRIZOL reagent according to the manufacturer's protocol, and quantified by measurement of optical density at 260 nm. Ratios of absorption (260/280 nm) of all preparations were between 1.8 and 2.0. Aliquots of RNA samples were subjected to electrophoresis in a 1% ethidium bromide stained 1.4% agarose formaldehyde gel to verify their integrity. Reverse transcription was performed using 2 μg of total RNA: 5.0 μg 5×RTbufer, 1.0 μg 106 RT Random Primer (Promega, Belgium), 2 μl 256 dNTP (Promega, Belgium), 0.5 μl Multiscribe Reverse Transcriptase (Promega, Belgium), 0.2 μl RNase inhibitor (Promega, Belgium), and the addition of nuclear free water to final volume of 25 μl. Reaction system was run at 37°C for 60 min and 95°C for 5 min.

Quantitative real-time RT-PCR was performed in a 20 μl reaction buffer that included 10 μl SYBR GREEN, 0.4 μl ROX, 0.4 μl of forward primer, 0.4 μl of reverse primer, 2 μl of cDNA, and 6.8 μl ddH_2_O were incubated in a Strategene MX3000PTM Detection System (Applied Biosystems). The reaction mixture was subject to program to conduct one cycle (95°C for 30 s) and 40 cycles (9°C for 5 s and 60°C for 30 s). Each sample was assayed in duplicate and the *m*RNA expression levels of the target genes were standardized against β-actin. The results (fold changes) were expressed as 2^-ΔΔ^C(t) with ΔΔC(t) = [C(t) ij−C(t) β-actinj]−[C(t) i1−C(t) β-actin1], where Ct ij and C(t) β-actinj are the Ct for gene i and for β-actin j in a pool or a sample(named j)and where Ct i1 and C(t) β-actin1 are the Ct in pool 1 or sample l, expressed as the standard.

### Statistical analysis

Data were expressed as means with SEM, and were analyzed using the SPSS 17.0 statistical package (SPSS, Inc., Chicago, IL, USA), performed by GLM procedures for significant differences, comparing the effects of diet (choline), type (IUGR) and the interaction (Type × Choline) between them. If significant differences (P<0.05) were found in factors, the Duncan’s new multiple range test was used to compare the means.

## Results

### Growth performance

The data on the effects of IUGR and dietary choline on growth performance are shown in **Table 4**. At 23, 73, and 120 d of age, the IUGR pigs had significantly lower (*P*<0.05) BW than NBW pigs. At 200 d of age, a trend towards to decrease in the BW in IUGR pigs was found as compared with NBW pigs; However, dietary choline had a trend to increase the BW at the age of 73 d. It was also found that a significant increase in the BW of both NBW and IUGR pigs at 120 d of age. The BW of pigs fed diets with choline had no significant difference (*P*<0.05) as compared with the BW of pigs fed with the basal diets at 200 d. Furthermore, there was no interaction observed between the type (IUGR or NBW) and choline.

**Table 4:** Effects of IUGR and choline on growth performance in pigs.

Day	NBW(kg)		IUGR(kg)		SEM	P value		
	N	N+C	I	I+C		Type	Choline	Type×Choline
23d	6.359±0.377	5.689±0.437	4.132±0.223	4.159±0.323	1.178	<0.01	0.373	0.337
73d	26.64±0.469	23.24±1.177	19.82±1.340	19.19±1.238	3.662	<0.01	0.095	0.237
120d	62.77±0.783	58.50±1.600	54.01±1.747	47.89±3.174	6.243	<0.01	0.003	0.529
200d	109.8±5.072	114.8±1.250	106.3±1.702	102.6±4.749	7.972	0.053	0.853	0.258

*NBW* normal body weight, *IUGR* intrauterine growth retardation.

*N* Group NBW, *N+C* Group NBW+Choline, *I* Group IUGR, *I+C* Group IUGR+Choline.

*SEM* standard error of the means.

### Meat quality

As shown in **Table 5**, the drip loss after 24 h and 48 h were not affected by the type and choline supplementation in diets (P>0.05); However, pH1 and pH24 values in the IUGR pigs were significantly higher (*P*<0.05) than the NBW pigs. Compared with the NBW pigs, L* and C* values were significantly increased (*P*<0.05), and H* values of *LD* meat muscle samples were significantly decreased (*P*<0.05) in IUGR pigs. Furthermore, the diets supplemented with choline were significantly decreased (*P*<0.05) the pH1 value compared with the pigs fed with basal diet, and L* and C* values were also increased in pigs fed with choline dietary.

**Table 5:** Effects of IUGR and choline on meat quality traits of *longissimus dorsi* muscle in pigs.

Item		Groups				SEM	P value		
		N	N+C	I	I+C		Type	Choline	Type×Choline
Drip loss%	24h	2.188±0.155	2.128±0.110	2.477±0.207	2.289±0.222	0.347	0.232	0.501	0.726
	48h	3.987±0.351	4.716±0.130	4.365±0.405	4.054±0.439	0.321	0.564	0.695	0.166
pH value	1h	6.801±0.033	7.056±0.016	6.647±0.049	6.921±0.101	0.188	0.031	0.001	0.857
	24h	5.563±0.038	5.5760.050	5.483±0.027	5.490±0.030	0.079	0.044	0.789	0.954
Meat Color	L*	38.47±0.315	42.47±0.759	41.51±0.337	43.45±0.418	2.116	<0.01	<0.01	0.057
	H*	1.351±0.007	1.344±0.014	1.278±0.010	1.262±0.014	0.456	<0.01	0.319	0.669
	C*	6.903±0.117	8.508±0.044	8.139±0.319	8.691±0.290	0.582	<0.01	<0.01	0.037

*NBW* normal body weight, *IUGR* intrauterine growth retardation.

*N* Group NBW, *N+C* Group NBW+Choline, *I* Group IUGR, *I+C* Group IUGR+Choline.

*SEM* standard error of the means.

### O2•-, MDA, DPPH and ABTS•+ scavenging activities

As shown in **Table 6**, both IUGR and dietary choline had no significant influence on DPPH and ABTS^•+^ scavenging activities in *LD* meat samples of pigs at 200 d of age. The MDA levels of *LD* meat samples were significantly higher (*P*<0.05) in both IUGR and choline supplemented pigs. The O_2_^•-^scavenging activity in *LD* meat samples was not significant (*P*>0.05) in the IUGR pigs, however, choline supplementation significantly increased (*P*<0.05) O_2_^•-^ scavenging activity in meat samples in pigs at 200 d of age.

**Table 6:** Effects of IUGR and choline on O_2_^•-^, DPPH and ABTS^•+^ scavenging activities *longissimus dorsi* muscle in pigs.

Item	Groups				SEM	P value		
	N	N+C	I	I+C		Type	Choline	Type×Choline
MDAnmol/mgprot	0.372±0.007	0.545±0.024	0.456±0.008	0.582±0.031	0.092	0.011	<0.01	0.270
O2^•-^ scavenging (U/L)	0.253±0.018	0.328±0.009	0.217±0.026	0.322±0.130	0.055	0.239	0.001	0.487
ABTS^•+^scavenging%	200.74±0.538	201.11±0.304	200.98±0.689	201.73±0.067	0.847	0.426	0.305	0.731
DPPH^•^scavenging%	167.79±2.655	168.39±2.455	163.58±2.031	169.83±1.907	4.16	0.562	0.172	0.251

*NBW* normal body weight, *IUGR* intrauterine growth retardation.

*N* Group NBW, *N+C* Group NBW+Choline, *I* Group IUGR, *I+C* Group IUGR+Choline.

*MDA* malondialdehyde, *O*_*2*_^*•-*^ superoxide radical

*ABTS* ^*•+*^ 2,2'-Azinobis-(3-ethylbenzthiazoline-6-sulphonate) radical cation

*DPPH* ^*•*^ 1,1-Diphenyl- 2-picrylhydrazyl radical 2,2-Diphenyl-1-(2,4,6-trinitrophenyl)hydrazyl

*SEM* standard error of the means.

### TG and IMF contents

The results of TG and IMF contents of *LD* meat samples in pigs are shown in **Table 7**. A significant increased (*P*<0.05) in TG and IMF contents were observed in the IUGR pig as compared with NBW pigs, while choline supplementation had a trend to reduce IMF content (*P* = 0.082) but significantly decreased (*P*<0.05) TG content. Furthermore, there was no interaction between type and choline supplementation in both TG and IMF contents in pigs at 200 d of age.

**Table 7:** Effects of IUGR and choline on TG and IMF content of *longissimus dorsi* muscle in pigs.

Item	Groups				SEM	P value		
	N	N+C	I	I+C		Type	Choline	Type×Choline
TG(mmol/L)	0.248±0.014	0.166±0.005	0.267±0.009	0.207±0.015	0.045	0.024	<0.01	0.540
IMF%	1.455±0.022	1.430±0.011	1.515±0.012	1.480±0.016	0.043	0.005	0.082	0.757

*NBW* normal body weight, *IUGR* intrauterine growth retardation.

*N* Group NBW, *N+C* Group NBW+Choline, *I* Group IUGR, *I+C* Group IUGR+Choline.

*TG* triglyceride, *IMF* intramuscular fat.

*SEM* standard error of the means.

### Intramuscular fat related genes mRNA expression

Fat metabolism *m*RNA gene expressions of *LD* meat muscle samples are given in **Table 8**. The ACC *m*RNA gene expression of IUGR pigs had a trend to an increase (P = 0.059) as compared with the NBW pigs at 200 d of age, and SREBP1 *m*RNA gene expressions of the IUGR pigs were significantly up-regulated (*P*<0.05) compared with the NBW pigs. As for the FAS *m*RNA gene expressions, Group I was significantly higher (*P*<0.05) than Group N, while there were no different effect between Group I+C and Group N (*P*>0.05). When diets supplemented with the choline, M-CPT *m*RNA gene expression of Group I+C was significantly increased (*P*<0.05) compared with the Group N and Group I. There was no significant difference in PPARγ *m*RNA expression among the experimental groups (data not shown). Additionally, there was an interaction effects on the IUGR and choline in FAS, M-CPT *m*RNA gene expressions (*P*<0.05).

**Table 8:** Effects of IUGR and choline on fat metabolism related genes of *longissimus dorsi* muscle in pigs.

Item	NBW		IUGR		SEM	P value		
	N	N+C	I	I+C		Type	Choline	Type×Choline
FAS	1.002±0.041^a^	0.434±0.032^b^	1.119±0.024^c^	1.003±0.041^a^	0.300	<0.01	<0.01	<0.01
ACC	1.004±0.061	0.540±0.027	1.367±0.143	0.547±0.042	0.383	0.059	<0.01	0.056
M-CPT	1.001±0.027^ab^	1.005±0.136^b^	0.760±0.064^a^	1.650±0.057^c^	0.363	0.061	<0.01	0.001
SREBP1	1.003±0.054	0.794±0.089	1.132±0.096	0.819±0.022	0.245	0.042	0.001	0.073

*NBW* normal body weight, *IUGR* intrauterine growth retardation.

*N* Group NBW, *N+C* Group NBW+Choline, *I* Group IUGR, *I+C* Group IUGR+Choline.

*FAS* fatty acid synthetase, *M-CPT1* muscle-carnitine palmityl transferase 1, *ACC* acetyl-CoA carboxylase, *SREBP1* sterol regulatory element-binding proteins.

*SEM* standard error of the means.

## Discussion

IUGR has become a significant problem in an intensive breeding system which presents high fertility and litter size [27,28]. Although the risk of obesity is mainly caused by large and rapidly growing factors, it appears to be measured whether IUGR newborns are related to the programmed obesity [29,30]. Previous studies had emphasized that the prevention of IUGR was crucial for increasing the efficiency of animal production [31]. Recently, growing interest in nutrition regulation to prohibit occurrence of IUGR pigs have taken more attention [32–34].

In the present study, the IUGR pigs had significantly lower BW than the NBW pigs. The results of the present study suggested that the IUGR had a permanent stunting effect on growth performance in pigs, which was in coincidence with the reports that IUGR pigs had lower BW [35,36]. The diets supplemented with the choline found that the growth performance was significantly improved at 120 d of age, which is in agreement with the previous studies in rats [37,38]. Our research also found that the effects of IUGR had a significant decrease in pH1 and pH24 values in *LD* meat muscles. It might be due to oxidative stress, is prone to lead fatty acid rancidity, which has a negative effect on pH value in meat quality [39,40]. However, the most interesting results were found that the IUGR pigs maintained higher L*, lower C*, and higher H* values in *LD* meat muscles. These color scales represented better meat color and the results might be related to the high content IMF in IUGR pigs. In the current study, our results found that the choline supplementation had a positive effect on meat quality and intramuscular fat metabolism. pH values were also significantly decreased, regulated by choline and the meat color had better values as well.

It is reported that when the lightest and heaviest birth weight pigs were analyzed at slaughter weight (mean weight of 111.8 kg) and the lightest littermates were found to contain higher levels of intramuscular lipid content compared with the heaviest littermate [41]. Similarly, in the current study, the TG and IMF contents of *LD* muscle in IUGR pigs were significantly higher than the NBW pigs. These results may be associated with the appetite enhancing the orexigenic mechanisms [30]. Furthermore, due to the low intramuscular protein levels in the IUGR pigs, the protein synthesis is reduced, and amino acids are directed to oxidation and fatty acid synthesis [42]. Consequently, these changes are expected to decrease protein accretion and increase fat deposition in skeletal muscle of the IUGR pigs [43]. It is known that obesity may induce systemic oxidative stress and that increased lipid peroxidation in accumulated fat, which may disturb the lipid bilayers of cell membrane, alter the membrane permeability, disrupt ionic channels and eventually lead to dysfunction of the cells especially whole erythrocytes [9]. Moreover, the muscle TG content was significantly down-regulated by choline supplementation in pig diets. There was a slight decrease in the IMF content of the *LD* muscle in the IUGR pigs. The previous reports found that the dietary choline could reduce the fat deposition by facilitating the fatty acid oxidation [44,45].

In this present study, the lipid peroxidation content was assessed by measuring the formation of MDA content, a well-known carbonyl product of oxidative lipid damage. The O2^•-^, one of the most representative free radical agent, is normally formed through a number of enzyme systems or non-enzymatic electron transfers and its effects can be magnified because it produces other kinds of cell-damaging free radicals and oxidizing molecules [46]. In our present study, the results found that the IUGR pigs had significantly higher MDA content compared with NBW pigs. The results of our present study are in agreement with recent studies suggesting that systemic oxidative stress correlates with body mass index [47]. Therefore, our results found increased oxidative stress in IMF and it was related to increased lipid peroxidation.

We determined the expression of genes and other factors, such as FAS, ACC M-CPT1 and their regulated factors, SREBP1 and PPARγ, which are involved in an increase of lipogenesis and the release of fatty acids. These results can be explained by the *m*RNA gene expressions of FAS and ACC in IMF, which are responsible for the fatty acids synthesis. They were all up-regulated by the effects of IUGR pigs at 200 d of age. The SREBP1 factor is the key chemical to regulate lipogenesis genes, such as FAS and ACC. In our present results, both the FAS and ACC *m*RNA gene expressions were increased in the IUGR pigs. However, M-CPT *m*RNA expression, the limited enzyme for fatty acid oxidation [18], was down-regulated in the IUGR pigs. These results are in agreement with the results in rats that the key enzymes are altered by the effects of IUGR, which is significantly increased in ACC *m*RNA gene expression and have a significant decreased in M-CPT *mRNA* gene expression in the IUGR rats. Moreover, a previous study found that the fat deposition in the IUGR pigs may be involved in energy utilization [12]. However, dietary choline appeared to down-regulate FAS and ACC *m*RNA gene expressions, as well as their regulators SREBP1 and M-CPT was up-regulated significantly in the Group I+C. Hence, dietary choline could improve the fat deposition by enhancing the lipogenesis and reducing the lipolysis. Previous studies also reported that the choline injection in normal rats increased plasma epinephrine concentrations which stimulates glycogen breakdown in muscle leading to the lipolysis [48]. However, other reports about the metabolism of choline indicated that it could promote the lipogenesis rather than inhibit fat deposition [49]. In addition, a study suggested that the dietary choline decreased the lipid content, can be attributed to an elevated incorporation of preexisting fatty acid into TG rather than *de novo* fatty acid synthesis, because the FAS *m*RNA gene expression remained the on same concentration in muscle cells by choline deficiency [22].

It is interesting to find that the MDA content and O_2_^•¯^ scavenging activity were significantly increased by the high concentration of choline regulation, nevertheless, some study found that the dietary choline restriction increased H_2_O_2_ generation in liver mitochondria also, markers of oxidative stress were elevated in the high-fat mice [50], while choline supplementation normalized the expression of the genes related with oxidative stress [51]. However, in our present study, the redox including both the oxidation and anti-oxidation were enhanced in the pigs supplemented with the choline, this may be related to the effect of high concentration of choline on IMF content, which is to mobilize fatty acids from TG stores toward mitochondrial uptake, causing enhanced mitochondrial fatty acid uptake.

## Conclusion

IUGR have a stunting growth and development, as well as causing fat deposition and lipid peroxidation in meat muscle in pigs. While, the supplementation of higher dietary levels of choline could reduce the fat deposition in muscle in pigs. This may suggest the potential use of choline as treatment to prevent fat accretion in the muscles even in humans.

## References

[1] Symonds ME, Mendez MA, Meltzer HM, Koletzko B, Godfrey K, Forsyth S, et al Early life nutritional programming of obesity: mother-child cohort studies. Annals of nutrition & metabolism. 2013;62(2):137–45. Epub 2013/02/09. 10.1159/000345598 . 23392264

[2] Neitzke U, Harder T, Plagemann A. Intrauterine growth restriction and developmental programming of the metabolic syndrome: a critical appraisal. Microcirculation (New York, NY: 1994). 2011;18(4):304–11. Epub 2011/03/23. 10.1111/j.1549-8719.2011.00089.x . 21418379

[3] Wu G, Bazer FW, Wallace JM, Spencer TE. Board-invited review: intrauterine growth retardation: implications for the animal sciences. Journal of animal science. 2006;84(9):2316–37. Epub 2006/08/16. 10.2527/jas.2006-156 . 16908634

[4] Meurens F, Summerfield A, Nauwynck H, Saif L, Gerdts V. The pig: a model for human infectious diseases. Trends in microbiology. 2012;20(1):50–7. Epub 2011/12/14. 10.1016/j.tim.2011.11.002 . 22153753 PMC7173122

[5] Miller ER, Ullrey DE. The pig as a model for human nutrition. Annual review of nutrition. 1987;7:361–82. Epub 1987/01/01. 10.1146/annurev.nu.07.070187.002045 . 3300739

[6] Jangjoo A, Mehrabi Bahar M, Aliakbarian M. Uncut Roux-en-y esophagojejunostomy: A new reconstruction technique after total gastrectomy. The Indian journal of surgery. 2010;72(3):236–9. Epub 2010/06/01. 10.1007/s12262-010-0059-7 ; PubMed Central PMCID: PMCPmc3452651. 23133254 PMC3452651

[7] Darragh AJ, Moughan PJ. The three-week-old piglet as a model animal for studying protein digestion in human infants. Journal of pediatric gastroenterology and nutrition. 1995;21(4):387–93. Epub 1995/11/01. PubMed . 8583289 10.1097/00005176-199511000-00004

[8] Germani D, Puglianiello A, Cianfarani S. Uteroplacental insufficiency down regulates insulin receptor and affects expression of key enzymes of long-chain fatty acid (LCFA) metabolism in skeletal muscle at birth. Cardiovascular diabetology. 2008;7:14 Epub 2008/05/20. 10.1186/1475-2840-7-14 ; PubMed Central PMCID: PMCPmc2396605. 18485240 PMC2396605

[9] Furukawa S, Fujita T, Shimabukuro M, Iwaki M, Yamada Y, Nakajima Y, et al Increased oxidative stress in obesity and its impact on metabolic syndrome. Journal of Clinical Investigation. 2004;114(12):1752–61. 15599400 10.1172/JCI21625 PMC535065

[10] Fujita K, Nishizawa H, Funahashi T, Shimomura I, Shimabukuro M. Systemic oxidative stress is associated with visceral fat accumulation and the metabolic syndrome. Circulation journal: official journal of the Japanese Circulation Society. 2006;70(11):1437–42. 17062967 10.1253/circj.70.1437

[11] Arsenijevic D, Onuma H, Pecqueur C, Raimbault S, Manning BS, Miroux B, et al Disruption of the uncoupling protein-2 gene in mice reveals a role in immunity and reactive oxygen species production. Nature genetics. 2000;26(4):435–9. 11101840 10.1038/82565

[12] Puglianiello A, Germani D, Antignani S, Tomba GS, Cianfarani S. Changes in the expression of hypothalamic lipid sensing genes in rat model of intrauterine growth retardation (IUGR). Pediatric research. 2007;61(4):433–7. Epub 2007/05/23. 10.1203/pdr.0b013e3180332d4e . 17515867

[13] Zeisel SH. Dietary choline: biochemistry, physiology, and pharmacology. Annual review of nutrition. 1981;1:95–121. Epub 1981/01/01. 10.1146/annurev.nu.01.070181.000523 . 6764726

[14] Pesti GM, Harper AE, Sunde ML. Choline/methionine nutrition of starting broiler chicks. Three models for estimating the choline requirement with economic considerations. Poultry science. 1980;59(5):1073–81. Epub 1980/05/01. PubMed . 7393840 10.3382/ps.0591073

[15] Niculescu MD, Zeisel SH. Diet, methyl donors and DNA methylation: interactions between dietary folate, methionine and choline. The Journal of nutrition. 2002;132(8 Suppl):2333s–5s. Epub 2002/08/07. PubMed . 12163687 10.1093/jn/132.8.2333S

[16] Niculescu MD, Craciunescu CN, Zeisel SH. Dietary choline deficiency alters global and gene-specific DNA methylation in the developing hippocampus of mouse fetal brains. FASEB journal: official publication of the Federation of American Societies for Experimental Biology. 2006;20(1):43–9. Epub 2006/01/06. 10.1096/fj.05-4707com ; PubMed Central PMCID: PMCPmc1635129. 16394266 PMC1635129

[17] Mehta AK, Singh BP, Arora N, Gaur SN. Choline attenuates immune inflammation and suppresses oxidative stress in patients with asthma. Immunobiology. 2010;215(7):527–34. Epub 2009/11/10. 10.1016/j.imbio.2009.09.004 . 19897276

[18] Wu G, Zhang L, Li T, Zuniga A, Lopaschuk GD, Li L, et al Choline supplementation promotes hepatic insulin resistance in phosphatidylethanolamine N-methyltransferase-deficient mice via increased glucagon action. The Journal of biological chemistry. 2013;288(2):837–47. Epub 2012/11/28. 10.1074/jbc.M112.415117 ; PubMed Central PMCID: PMCPmc3543033. 23179947 PMC3543033

[19] Pacelli C, Coluccia A, Grattagliano I, Cocco T, Petrosillo G, Paradies G, et al Dietary choline deprivation impairs rat brain mitochondrial function and behavioral phenotype. The Journal of nutrition. 2010;140(6):1072–9. Epub 2010/04/02. 10.3945/jn.109.116673 . 20357080

[20] Schugar RC, Huang X, Moll AR, Brunt EM, Crawford PA. Role of choline deficiency in the Fatty liver phenotype of mice fed a low protein, very low carbohydrate ketogenic diet. PloS one. 2013;8(8):e74806 Epub 2013/09/07. 10.1371/journal.pone.0074806 ; PubMed Central PMCID: PMCPmc3756977. 24009777 PMC3756977

[21] Ossani GP, Repetto MG, Boveris A, Monserrat AJ. The protective effect of menhaden oil in the oxidative damage and renal necrosis due to dietary choline deficiency. Food & function. 2013;4(3):448–52. Epub 2012/12/14. 10.1039/c2fo30229b . 23235886

[22] Michel V, Singh RK, Bakovic M. The impact of choline availability on muscle lipid metabolism. Food & function. 2011;2(1):53–62. Epub 2011/07/21. 10.1039/c0fo00069h . 21773586

[23] Xu RJ, Mellor DJ, Birtles MJ, Reynolds GW, Simpson HV. Impact of intrauterine growth retardation on the gastrointestinal tract and the pancreas in newborn pigs. Journal of pediatric gastroenterology and nutrition. 1994;18(2):231–40. Epub 1994/02/01. PubMed . 8014773 10.1097/00005176-199402000-00018

[24] Beijing animal husbandry and veterinary institute, Chinese academy of agricultural sciences. Database of China Feed. Available: http://www.chinafeeddata.org.cn/.

[25] Re R, Pellegrini N, Proteggente A, Pannala A, Yang M, Rice-Evans C. Antioxidant activity applying an improved ABTS radical cation decolorization assay. Free radical biology & medicine. 1999;26(9–10):1231–7. Epub 1999/06/25. PubMed . 10381194 10.1016/s0891-5849(98)00315-3

[26] Cheng Z, Moore J, Yu L. High-throughput relative DPPH radical scavenging capacity assay. Journal of agricultural and food chemistry. 2006;54(20):7429–36. Epub 2006/09/28. 10.1021/jf0611668 . 17002404

[27] Rosenberg A. The IUGR newborn. Seminars in perinatology. 2008;32(3):219–24. Epub 2008/05/17. 10.1053/j.semperi.2007.11.003 . 18482625

[28] Michel CL, Bonnet X. Influence of body condition on reproductive output in the guinea pig. Journal of experimental zoology Part A, Ecological genetics and physiology. 2012;317(1):24–31. Epub 2011/11/08. 10.1002/jez.714 . 22058022

[29] Desai M, Ross MG. Fetal programming of adipose tissue: effects of intrauterine growth restriction and maternal obesity/high-fat diet. Seminars in reproductive medicine. 2011;29(3):237–45. Epub 2011/06/29. 10.1055/s-0031-1275517 ; PubMed Central PMCID: PMCPmc4010300. 21710399 PMC4010300

[30] Fukami T, Sun X, Li T, Desai M, Ross MG. Mechanism of programmed obesity in intrauterine fetal growth restricted offspring: paradoxically enhanced appetite stimulation in fed and fasting states. Reproductive sciences (Thousand Oaks, Calif). 2012;19(4):423–30. Epub 2012/02/22. 10.1177/1933719111424448 ; PubMed Central PMCID: PMCPmc3343065. 22344733 PMC3343065

[31] Redmer DA, Wallace JM, Reynolds LP. Effect of nutrient intake during pregnancy on fetal and placental growth and vascular development. Domestic animal endocrinology. 2004;27(3):199–217. Epub 2004/09/29. 10.1016/j.domaniend.2004.06.006 . 15451070

[32] Wu G, Bazer FW, Burghardt RC, Johnson GA, Kim SW, Li XL, et al Impacts of amino acid nutrition on pregnancy outcome in pigs: mechanisms and implications for swine production. Journal of animal science. 2010;88(13 Suppl):E195–204. Epub 2009/10/27. 10.2527/jas.2009-2446 . 19854987

[33] Wu G, Bazer FW, Cudd TA, Meininger CJ, Spencer TE. Maternal nutrition and fetal development. The Journal of nutrition. 2004;134(9):2169–72. Epub 2004/08/31. PubMed . 15333699 10.1093/jn/134.9.2169

[34] Desai M, Gayle D, Babu J, Ross MG. Programmed obesity in intrauterine growth-restricted newborns: modulation by newborn nutrition. American journal of physiology Regulatory, integrative and comparative physiology. 2005;288(1):R91–6. Epub 2004/08/07. 10.1152/ajpregu.00340.2004 . 15297266

[35] Kenney JL, Carlberg KA. The effect of choline and myo-inositol on liver and carcass fat levels in aerobically trained rats. International journal of sports medicine. 1995;16(2):114–6. Epub 1995/02/01. 10.1055/s-2007-972975 . 7751073

[36] Merlet-Benichou C, Gilbert T, Muffat-Joly M, Lelievre-Pegorier M, Leroy B. Intrauterine growth retardation leads to a permanent nephron deficit in the rat. Pediatric nephrology (Berlin, Germany). 1994;8(2):175–80. Epub 1994/04/01. PubMed . 8018495 10.1007/BF00865473

[37] Saunderson CL, Mackinlay J. Changes in body-weight, composition and hepatic enzyme activities in response to dietary methionine, betaine and choline levels in growing chicks. The British journal of nutrition. 1990;63(2):339–49. Epub 1990/03/01. PubMed . 1692235 10.1079/bjn19900120

[38] Hongu N, Sachan DS. Caffeine, carnitine and choline supplementation of rats decreases body fat and serum leptin concentration as does exercise. The Journal of nutrition. 2000;130(2):152–7. Epub 2000/03/17. PubMed . 10720162 10.1093/jn/130.2.152

[39] Peterside IE, Selak MA, Simmons RA. Impaired oxidative phosphorylation in hepatic mitochondria in growth-retarded rats. American journal of physiology Endocrinology and metabolism. 2003;285(6):E1258–66. Epub 2003/11/11. 10.1152/ajpendo.00437.2002 . 14607783

[40] Selak MA, Storey BT, Peterside I, Simmons RA. Impaired oxidative phosphorylation in skeletal muscle of intrauterine growth-retarded rats. American journal of physiology Endocrinology and metabolism. 2003;285(1):E130–7. Epub 2003/03/15. 10.1152/ajpendo.00322.2002 . 12637257

[41] Karunaratne JF, Ashton CJ, Stickland NC. Fetal programming of fat and collagen in porcine skeletal muscles. Journal of anatomy. 2005;207(6):763–8. Epub 2005/12/22. 10.1111/j.1469-7580.2005.00494.x ; PubMed Central PMCID: PMCPmc1571588. 16367803 PMC1571588

[42] Wang J, Chen L, Li D, Yin Y, Wang X, Li P, et al Intrauterine growth restriction affects the proteomes of the small intestine, liver, and skeletal muscle in newborn pigs. The Journal of nutrition. 2008;138(1):60–6. Epub 2007/12/25. PubMed . 18156405 10.1093/jn/138.1.60

[43] Lecker SH, Jagoe RT, Gilbert A, Gomes M, Baracos V, Bailey J, et al Multiple types of skeletal muscle atrophy involve a common program of changes in gene expression. FASEB journal: official publication of the Federation of American Societies for Experimental Biology. 2004;18(1):39–51. Epub 2004/01/14. 10.1096/fj.03-0610com . 14718385

[44] Tryndyak V, de Conti A, Kobets T, Kutanzi K, Koturbash I, Han T, et al Interstrain differences in the severity of liver injury induced by a choline- and folate-deficient diet in mice are associated with dysregulation of genes involved in lipid metabolism. FASEB journal: official publication of the Federation of American Societies for Experimental Biology. 2012;26(11):4592–602. Epub 2012/08/09. 10.1096/fj.12-209569 ; PubMed Central PMCID: PMCPmc3475259. 22872676 PMC3475259

[45] Best CH, Huntsman ME. The effect of choline on the liver fat of rats in various states of nutrition. The Journal of physiology. 1935;83(3):255–74. Epub 1935/02/09. PubMed ; PubMed Central PMCID: PMCPmc1394528. 16994629 10.1113/jphysiol.1935.sp003227 PMC1394528

[46] Dudonne S, Vitrac X, Coutiere P, Woillez M, Merillon JM. Comparative study of antioxidant properties and total phenolic content of 30 plant extracts of industrial interest using DPPH, ABTS, FRAP, SOD, and ORAC assays. Journal of agricultural and food chemistry. 2009;57(5):1768–74. Epub 2009/02/10. 10.1021/jf803011r . 19199445

[47] Keaney JF, Larson MG, Vasan RS, Wilson PW, Lipinska I, Corey D, et al Obesity and systemic oxidative stress clinical correlates of oxidative stress in the Framingham Study. Arteriosclerosis, Thrombosis, and Vascular Biology. 2003;23(3):434–9. 12615693 10.1161/01.ATV.0000058402.34138.11

[48] Wu P, Jiang J, Liu Y, Hu K, Jiang WD, Li SH, et al Dietary choline modulates immune responses, and gene expressions of TOR and eIF4E-binding protein2 in immune organs of juvenile Jian carp (Cyprinus carpio var. Jian). Fish & shellfish immunology. 2013;35(3):697–706. Epub 2013/06/19. 10.1016/j.fsi.2013.05.030 . 23774323

[49] Fernandez-Figares I, Lachica M, Martin A, Nieto R, Gonzalez-Valero L, Rodriguez-Lopez JM, et al Impact of dietary betaine and conjugated linoleic acid on insulin sensitivity, protein and fat metabolism of obese pigs. Animal: an international journal of animal bioscience. 2012;6(7):1058–67. Epub 2012/10/04. 10.1017/s1751731111002308 . 23031465

[50] Hensley K, Kotake Y, Sang H, Pye QN, Wallis GL, Kolker LM, et al Dietary choline restriction causes complex I dysfunction and increased H2O2 generation in liver mitochondria. Carcinogenesis. 2000;21(5):983–9. 10783322 10.1093/carcin/21.5.983

[51] Sachan DS, Hongu N, Johnsen M. Decreasing oxidative stress with choline and carnitine in women. Journal of the American College of Nutrition. 2005;24(3):172–6. 15930482 10.1080/07315724.2005.10719462

